# Optimising postoperative surveillance for hepatocellular carcinoma: Beyond histological predictors^☆^

**DOI:** 10.1016/j.iliver.2024.100110

**Published:** 2024-08-11

**Authors:** Jiahao Xu, Lihui Gu, Tian Yang

**Affiliations:** a Department of Hepatobiliary Surgery, Eastern Hepatobiliary Surgery Hospital, Naval Medical University, Shanghai 200438, China; b Graduate School, Bengbu Medical University, Bengbu 233000, China

We read with great interest the recent publication by Fuster-Anglada et al. [1], which investigated the histological predictors of aggressive recurrence of hepatocellular carcinoma (HCC) following liver resection. The study provides valuable insights into the prognostic significance of microvascular invasion and other histological features, which are instrumental for guiding clinical decision-making and patient follow-up after surgery. While the authors have thoroughly detailed the standard imaging follow-up schedule, we believe that several critical aspects warrant further discussion.

First, the practical issue of patient adherence to the proposed follow-up regimen remains unaddressed. In real-world clinical practice, non-adherence to regular follow-up protocols can lead to a delayed diagnosis of recurrence, potentially allowing for more aggressive tumour progression. Our team previously demonstrated that no/irregular recurrence surveillance is an independent risk factor for beyond-Milan recurrence, *i.e.*, aggressive recurrence, after hepatectomy for early-stage HCC (HR: 1.70) [2]. Patients undergoing regular surveillance had significantly lower 5-year cumulative rates of aggressive recurrence compared with those with no/irregular surveillance (17.6% *vs.* 25.7%, *p* = 0.007).

Second, the impact of surveillance on treatment options cannot be overstated. Our research found that only 21.7% of patients with aggressive recurrence ultimately received potentially curative treatments compared with 62.7% of patients with within-Milan recurrence (*p* < 0.001) [2]. This stark difference highlights the critical importance of early detection through regular surveillance in preserving treatment options. Furthermore, in another study on late recurrence after HCC resection, we found that adherence to regular surveillance was significantly associated with the increased chance of receiving curative treatment for late recurrence (*p* = 0.001) and improved post-recurrence survival through potentially curative treatments (*p* < 0.001) [3].

Lastly, while regular surveillance is crucial, the cost-effectiveness of different surveillance strategies needs to be considered, especially in resource-limited settings. Future studies should address the balance between surveillance intensity and resource utilisation to optimize patient outcomes while maintaining economic feasibility.

To enhance the effectiveness of postoperative surveillance, we recommend that future studies consider the following:

1) Assessing patient-reported barriers to follow-up care and implementing strategies to enhance patient engagement, such as reminder systems or telemedicine consultations.
2) Incorporating patient feedback to tailor follow-up schedules to individual needs and circumstances.
3) Evaluating the cost-effectiveness of different surveillance strategies to inform policy decisions and resource allocation.
4) Investigating the potential role of liquid biopsy or other novel biomarkers in complementing imaging-based surveillance [4,5].

In conclusion, we commend the authors for their significant contribution to the field and hope this correspondence will stimulate further discussion on optimizing postoperative surveillance for HCC. By addressing these additional aspects, we can work towards a more comprehensive and patient-centred approach to postoperative care in HCC.

## Funding

The authors received no financial support to produce this manuscript.

## CRediT authorship contribution statement

**Jiahao Xu:** Conceptualization, Writing – original draft. **Lihui Gu:** Conceptualization, Writing – original draft. **Tian Yang:** Conceptualization, Writing – original draft, Writing – review & editing. Jiahao Xu and Lihui Gu contributed equally to this commentary.

## Data available statement

Not applicable.

## Ethics statement

Not applicable.

## Informed consent

Not applicable.

## Declaration of competing interest

All authors have declared no conflict of interest.
